# Supplementary material for: Fragmentation and multithreading of experience in the default-mode network
Source: Nat Commun. 2025 Sep 25;16:8401. doi: 10.1038/s41467-025-63522-y (PMC12462485; doi:10.1038/s41467-025-63522-y)
Supplement: Supplementary file 2 — Reporting Summary [file 41467_2025_63522_MOESM2_ESM.pdf]

nature portfolio

Corresponding author(s): Fahd Yazin
Last updated by author(s): Jul 9, 2025

Reporting Summary

Nature Portfolio wishes to improve the reproducibility of the work that we publish. This form provides structure for consistency and transparency in reporting. For further information on Nature Portfolio policies, see our Editorial Policies and the Editorial Policy Checklist.

Statistics

For all statistical analyses, confirm that the following items are present in the figure legend, table legend, main text, or Methods section.

n/a	Confirmed	
☐	☒	The exact sample size (*n*) for each experimental group/condition, given as a discrete number and unit of measurement
☐	☒	A statement on whether measurements were taken from distinct samples or whether the same sample was measured repeatedly
☐	☒	The statistical test(s) used AND whether they are one- or two-sided<br>*Only common tests should be described solely by name; describe more complex techniques in the Methods section.*
☐	☒	A description of all covariates tested
☐	☒	A description of any assumptions or corrections, such as tests of normality and adjustment for multiple comparisons
☐	☒	A full description of the statistical parameters including central tendency (e.g. means) or other basic estimates (e.g. regression coefficient) AND variation (e.g. standard deviation) or associated estimates of uncertainty (e.g. confidence intervals)
☐	☒	For null hypothesis testing, the test statistic (e.g. *F*, *t*, *r*) with confidence intervals, effect sizes, degrees of freedom and *P* value noted<br>*Give P values as exact values whenever suitable.*
☐	☒	For Bayesian analysis, information on the choice of priors and Markov chain Monte Carlo settings
☐	☒	For hierarchical and complex designs, identification of the appropriate level for tests and full reporting of outcomes
☐	☒	Estimates of effect sizes (e.g. Cohen's *d*, Pearson's *r*), indicating how they were calculated

Our web collection on statistics for biologists contains articles on many of the points above.

Software and code

Policy information about availability of computer code

Data collection	The movie neuroimaging data was sourced from the open-source CAMCAN database (Shafto et a 2014l). The narrative neuroimaging data was sourced from open-source Narrative dataset (Nastase et al 2021). The belief-update time courses were obtained from Prolific online participants
Data analysis	All the code (including behavioural data) for replicating the analysis/results are in https://osf.io/bpvj4/

For manuscripts utilizing custom algorithms or software that are central to the research but not yet described in published literature, software must be made available to editors and reviewers. We strongly encourage code deposition in a community repository (e.g. GitHub). See the Nature Portfolio guidelines for submitting code & software for further information.

Data

Policy information about availability of data

All manuscripts must include a data availability statement. This statement should provide the following information, where applicable:

- Accession codes, unique identifiers, or web links for publicly available datasets
- A description of any restrictions on data availability
- For clinical datasets or third party data, please ensure that the statement adheres to our policy

Datasets are publicly available open-source

Research involving human participants, their data, or biological material

Policy information about studies with human participants or human data. See also policy information about sex, gender (identity/presentation), and sexual orientation and race, ethnicity and racism.

Reporting on sex and gender	*Use the terms sex (biological attribute) and gender (shaped by social and cultural circumstances) carefully in order to avoid confusing both terms. Indicate if findings apply to only one sex or gender; describe whether sex and gender were considered in study design; whether sex and/or gender was determined based on self-reporting or assigned and methods used. Provide in the source data disaggregated sex and gender data, where this information has been collected, and if consent has been obtained for sharing of individual-level data; provide overall numbers in this Reporting Summary. Please state if this information has not been collected. Report sex- and gender-based analyses where performed, justify reasons for lack of sex- and gender-based analysis.*
Reporting on race, ethnicity, or other socially relevant groupings	*Please specify the socially constructed or socially relevant categorization variable(s) used in your manuscript and explain why they were used. Please note that such variables should not be used as proxies for other socially constructed/relevant variables (for example, race or ethnicity should not be used as a proxy for socioeconomic status). Provide clear definitions of the relevant terms used, how they were provided (by the participants/respondents, the researchers, or third parties), and the method(s) used to classify people into the different categories (e.g. self-report, census or administrative data, social media data, etc.) Please provide details about how you controlled for confounding variables in your analyses.*
Population characteristics	Young, healthy adults aged 18-35years
Recruitment	Offline neuroimaging data was from CAMCAN (movie, UK based cohort) and Narrative dataset (Princeton cohort)<br>Online participants were demographically matched with the fMRI participants (UK residents aged 21-35)
Ethics oversight	The online rating studies were approved by University of Edinburgh School of Philosophy, Psychology & Language Sciences Research Ethics Committee and all participants gave written informed consent.

Note that full information on the approval of the study protocol must also be provided in the manuscript.

Field-specific reporting

Please select the one below that is the best fit for your research. If you are not sure, read the appropriate sections before making your selection.

☒ Life sciences ☐ Behavioural & social sciences ☐ Ecological, evolutionary & environmental sciences

For a reference copy of the document with all sections, see nature.com/documents/nr-reporting-summary-flat.pdf

Life sciences study design

All studies must disclose on these points even when the disclosure is negative.

Sample size	Movie fMRI (n=111), Narrative fMRI (n=52)<br>Belief updates + Arousal in both (n= 129)
Data exclusions	Original Movie fMRI cohort was 135, of which we removed 24 due to exceeding a maximum framewise displacement of 1 mm or angular rotation exceeding 1.5°. Of the 56 subjects in narrative dataset, we excluded two subjects as suggested in the original paper and excluded two more subjects because there was an issue with accessing the data.
Replication	Central results (Fragmentation & Multithreading) observed in the Movie were replicated in the Narrative with the exact same methods and statistical tests.
Randomization	Naturalistic stimuli which does not need randomisation
Blinding	NA

Reporting for specific materials, systems and methods

We require information from authors about some types of materials, experimental systems and methods used in many studies. Here, indicate whether each material, system or method listed is relevant to your study. If you are not sure if a list item applies to your research, read the appropriate section before selecting a response.

Materials & experimental systems

n/a	Involved in the study
☒	☐ Antibodies
☒	☐ Eukaryotic cell lines
☒	☐ Palaeontology and archaeology
☒	☐ Animals and other organisms
☒	☐ Clinical data
☒	☐ Dual use research of concern
☒	☐ Plants

Methods

n/a	Involved in the study
☒	☐ ChIP-seq
☒	☐ Flow cytometry
☐	☒ MRI-based neuroimaging

Plants

Seed stocks	NA
Novel plant genotypes	NA
Authentication	NA

Magnetic resonance imaging

Experimental design

Design type	Naturalistic neuroimaging a) Movie (audio+visual, black & white) b) Narrative (audio only)
Design specifications	Movie was 8m 13s long (193 TRs)<br>Narrative was 9m 7s long (400 TRs synced to stimuli onset)<br>No other tasks were included during these scanning runs as per their original publications
Behavioral performance measures	Instruction-free, Response-free, freely viewing paradigms<br>For more information related to whether behaviour collected in these studies (which are unreported in this manuscript), please refer to these works in the main text (Shafto et al, Nastase et al)

Acquisition

Imaging type(s)	Functional
Field strength	Movie (3T Siemens TIM Trio system), Narrative (3T Siemens Magnetom Skyra)
Sequence & imaging parameters	Movie: Multi-echo T2* Gradient Echo EPI, N=193 volumes of 32 axial slices 3.7mm thick, voxel size of 3x3x4.4mm, 0.74mm gap, TR=2470ms; TE=[9,4, 21.2, 33, 45, 57]ms, FA=78 deg; FOV =192 mm × 192 mm;Matrix size: 64x64<br><br>Narrative: Gradient-echo echo-planar imaging (EPI) with an in-plane acceleration factor of 2 using GRAPPA: TR/TE= 1500/28 ms, flip angle = 64°, in-plane resolution = 3×3 mm, slice thickness = 4 mm, matrix size = 64×64, FoV = 192×192 mm, 27 axial slices
Area of acquisition	Movie: Whole-brain<br>Narrative: Roughly whole brain coverage (In cases where full brain coverage was not attainable, inferior extremities were typically excluded (e.g. cerebellum, brainstem) to maximize coverage of the cerebral cortex.)

Diffusion MRI ☐ Used ☒ Not used

Preprocessing

Preprocessing software	Movie: Statistical Parametric Mapping (SPM 12) (The preprocessing was performed on raw signals and included spatial realignment, slice-timing correction, coregistration of functional (T2) to anatomical (T1) scans, affine transformation to Montreal Neurological Institute (MNI) template (MNI152), resampling to 2 mm isotropic voxels, and spatial smoothing with a 6 × 6 × 9-mm full-width at half-maximum Gaussian kernel)<br>Narrative: fMRIPrep 20.0.5 (https://www.nature.com/articles/s41597-021-01033-3) (Smoothing 8x8x8-mm)
Normalization	Movie: Affine transformation to Montreal Neurological Institute (MNI) template (MNI152)

Normalization	Narrative: The BOLD time-series were resampled into standard space and correspondingly generating the following spatially-normalized, preprocessed BOLD run: MNI152NLin6Asym.
Normalization template	Movie: Montreal Neurological Institute (MNI) template (MNI152)<br>Narrative: Volumetric normalization template MNI152NLin6Asym
Noise and artifact removal	Movie: Six motion parameters (interscan X-, Y-, Z-displacement and pitch, roll, and yaw parameters) were regressed out from functional data by least square regression. Subjects who had maximum framewise displacement >1 mm or 1.5° were discarded from further analysis. All functional data underwent voxel-wise detrending and was band-pass filtered in the range of 0.01–0.1 Hz using a second-order Butterworth filter.(Majumdar et al, 2023)<br>Narrative: Pre-processed time-series were calculated from the preprocessed BOLD data, including framewise displacement (FD), DVARS, and three global signals (CSF, WM, and whole-brain). FD and DVARS were computed for each functional run using Nipype implementations. Physiological noise correction was performed with component-based noise correction (CompCor), using both temporal (tCompCor) and anatomical (aCompCor) variants. The tCompCor components were derived from the top 5% most variable voxels within subcortical regions, after applying a 128-second high-pass filter to the BOLD time-series.
Volume censoring	Movie: Following preprocessing, the first 4 functional scans were discarded, which yielded a total of 189 scans for each subject.<br>Narrative: Not performed

Statistical modeling & inference

Model type and settings	Mass univariate (for main GLM) (Frequentist & Bayesian)<br>Bayesian hierarchical regression with hypothesis testing on posterior estimates (Rstan/brms) on GLM Betas, Intersubject correlation and Intersubject functional connectivity. Random effects over participants and ROIs (for model details, priors and predictive checks see the main/supplementary info)<br>Phase scrambled permutation test for significance testing on Hidden Markov Model (windows) and Intersubject pattern correlation time-course similarity between regions (correlation values)
Effect(s) tested	For frequentist analyses, effect sizes (Coh d) and p-values are computed<br>For Bayesian analyses, Bayes factors and posterior probability are used for effect size<br>For simulation-based/permutation analysis, p value under simulated null distribution (alpha 0.05) are used for assessing statistical effects

Specify type of analysis: ☐ Whole brain ☐ ROI-based ☒ Both

Anatomical location(s)	All prefrontal ROI masks were defined from the Brainnetome Atlas using combining subregions from each zone, and can be see in the supplmentary mats. Other ROIs were used from a previous study on the Movie data (Majumdar et al 2023)
Statistic type for inference (See Eklund et al. 2016)	GLM Statistical maps were thresholded at q < 0.05 FDR with an extent threshold of 25 voxels (k = 25)
Correction	FDR correction for the GLM maps<br>voxel-wise ISFC maps were thresholded at p<0.001 FDR and visualized at r > 0.1

Models & analysis

n/a	Involved in the study
☐	☒ Functional and/or effective connectivity
☒	☐ Graph analysis
☐	☒ Multivariate modeling or predictive analysis

Functional and/or effective connectivity	Pearson correlation for intersubject functional connectivity
Multivariate modeling and predictive analysis	Bayesian hierarchical regression were assessed by visually inspecting chains, Bayes R2 values (0.72/0.48/0.85/0.42) for the four models), R-hat values (~1.00) and posterior predictive checks (Supplementary mats)
